# Cell-free osteochondral scaffolds provide a substantial clinical benefit in the treatment of osteochondral defects at a minimum follow-up of 5 years

**DOI:** 10.1186/s40634-021-00381-8

**Published:** 2021-08-16

**Authors:** Martina Ricci, Daniele Tradati, Alessio Maione, Francesco Mattia Uboldi, Eva Usellini, Massimo Berruto

**Affiliations:** UOS Chirurgia Articolare del Ginocchio, I Clinica Ortopedica, ASST Gaetano Pini-CTO, Piazza Cardinal Ferrari 1, 20122 Milan, Italy

**Keywords:** Cartilage, Osteochondral lesion, Osteochondral substitute, Scaffold, Knee, Osteochondritis dissecans, Osteonecrosis, Biomimetic

## Abstract

**Purpose:**

The treatment of osteochondral lesions is challenging and no consensus has been established about the best option for restoring both cartilage and subchondral bone. Multilayer collagen-hydroxyapatite scaffolds have shown promising clinical results, but the outcome at a follow-up longer than 5 years still has to be proved. The aim was to evaluate the clinical outcome of patients with a knee isolated osteochondral lesion treated with a biomimetic three-layered scaffold at a minimum 5 years of follow-up.

**Methods:**

Twenty-nine patients (23 males and 6 females, mean age 31.5 ± 11.4 years) were evaluated retrospectively before surgery, at 1 and 2 years and at last follow-up (FU). Visual Analog Scale (VAS) for pain, International Knee Documentation Committee (IKDC) Subjective Score, Tegner-Lysholm Knee Scoring Scale and Tegner Activity Level Scale were collected. Mean FU was 7.8 ± 2.0 years (min 5.1 - max 11.3). The etiology of the defect was Osteochondritis Dissecans or osteonecrosis (17 vs 12 cases).

**Results:**

At 12 months FU the IKDC score improved from 51.1 ± 21.7 to 80.1 ± 17.9 (*p* < 0.01), Tegner Lysholm Score from 59.9 ± 17.3 to 92.5 ± 9.0 (*p* < 0.01), VAS from 6.1 ± 2.1 to 1.7 ± 2.3 (*p* < 0.01) and Tegner Activity Level Scale from 1.6 ± 0.5 to 4.9 ± 1.7 (*p* < 0.01). The results remained stable at 24 months, while at last FU a statistically significant decrease in IKDC, Tegner Lysholm and Tegner Activity Scale was recorded, though not clinically relevant. Patients under 35 achieved statistically better outcomes.

**Conclusions:**

The use of a cell-free collagen-hydroxyapatite osteochondral scaffold provides substantial clinical benefits in the treatment of knee osteochondral lesions at a minimum follow-up of 5 years, especially in patients younger than 35 years.

**Level of evidence:**

Level IV.

## Background

Articular cartilage represents a specialized tissue characterized by low repairing capacities. Lately, a growing interest has been addressed to the role of subchondral bone in the progression of cartilaginous damage as well as in the repair process. Indeed, mesenchymal stem cells from the subchondral bone have been considered the source of chondrocyte progenitors, responsible for new cartilage synthesis in case of damage. Up to now, different surgical techniques have been proposed for the treatment of knee osteochondral lesions [3]. These include Bone Marrow Stimulation techniques (BMS), aimed to carry MSCs from the subchondral bone to the lesion site, and different procedures intended to promote cartilage formation such as Autologous Chondrocyte Implantation (ACI) and Matrix-induced Autologous Chondrocyte Implantation (MACI). Osteochondral autografts (OATS) or allografts, instead, represent an option for restoring the entire osteochondral unit. Cell-free scaffolds have been proposed as a feasible single-step alternative for the treatment of osteochondral defects, not burdened by the disadvantages and risks of two-step surgeries, donor site morbidity or diseases transmission. In the last years, different osteochondral scaffolds have been developed: they can be composed by several biomaterials, like hyaluronic acid, collagen, hydroxyapatite, alginate, polylactide-glycolide copolymers, calcium-sulfate/phosphate; they can be also enhanced by growth factors, such as TGF-β3 and BMP-2, or even human MSCs or chondrocytes derived from nasal cartilage [44, 46]. Up to now, though, only few are available for clinical use. Good to excellent results have been reported by different authors using hyaluronic acid-based and chitosan-glycerol phosphate scaffolds [4, 38, 49]. In 30 patients, a biphasic polylactic-co-glycolic acid and calcium sulfate scaffold was employed showing good functional outcome and integration at MRI at 48 months [9]; the same system implanted arthroscopically was considered effective, with a low failure rate at 101 months follow-up in high grade osteochondral lesions, though imaging results were found to be controversial [7]. Chitosan-glycerol phosphate/blood implant combined to microfracture was compared to a hyaluronic acid-based cell-free scaffold revealing similar clinical and radiographic outcomes at short-term, thus it can represent an effective choice especially in lesion smaller than 3 cm^2^ [45]. Filtered bone marrow aspirate containing mesenchymal stem/stromal cells, was combined with a biomimetic collagen-hydroxyapatite scaffold and implanted in 15 chronic knee osteochondral lesions with a significant increase of all KOOS subscales at 20 months and nearly normal to normal cartilage repair in the 7/8 MRI or arthroscopic evaluations performed in the study by Veber et al. [48].

The main purpose of the current study was to evaluate the clinical outcome of patients affected by isolated osteochondral lesions of the knee who underwent implantation of a three-layered synthetic osteochondral scaffold (MaioRegen®, FinCeramica, Faenza, Italy), at a minimum follow-up of 5 years. This device is a multi-layered matrix made of equine collagen and magnesium-enriched hydroxyapatite, characterized by a biomimetic porous structure meant to favour cellular colonization and differentiation in order to regenerate both bone and cartilage layers of the osteochondral defect. In our hypothesis patients undergoing scaffold implantation would result in improved clinical scores in comparison to the preoperative values even at mid-term follow-up.

## Methods

### Study design and patient selection

In this retrospective study, a consecutive series of 54 patients who underwent osteochondral scaffold implantation by a single orthopedic surgeon between December 2008 and June 2019 was evaluated. Patients were considered eligible for inclusion in the study if they (1) underwent an isolated scaffold implantation procedure, (2) had a full clinical evaluation pre-operatively and then 12 and 24 months post-operatively, (3) had a minimum follow-up of 5 years and (4) gave signed informed consent to their participation in the study.

Patients were excluded if they met one of the following criteria: (1) preoperative tibio-femoral Osteoarthritis (*Kellgren–Lawrence* grade 3–4) [19], (2) previous surgical cartilage procedures addressed to the same lesion, (3) presence of any contraindications to the scaffold implantation.

### Data collection

Preoperative and intraoperative findings were extracted from the internal database of the institution. The following data were recorded for each patient: gender, age at the time of surgery, BMI, etiology, location and size of the lesion. Regarding the aetiology of the osteochondral defect, lesions were classified as Osteochondritis dissecans (OCD) or Osteonecrosis (ON). OCD is a pathologic process in which the subchondral bone and the overlying articular cartilage detach from the underlying bony surface: the process originally starts deep underneath the articular surface and subsequently involves the articular cartilage at the peripheral border of the lesion with an “inside-out” mechanism. The cause is still unknown but is likely related to repetitive microtrauma, ischemia, an ossification abnormality, or endocrine or genetic predisposition; it mainly affects pediatric and adolescent patients on the lateral aspect of medial femoral condyle [14, 40]. Osteonecrosis is the result of a reduction or complete loss of blood supply to the bone and can be encountered in epiphyseal or subarticular area, leading to a collapse of the articular surface. It can be idiopathic, but common causes of osteonecrosis include trauma, use of corticosteroids, sickle cell anemia, collagen vascular disease, and alcoholism. It tends to develop in adults, most commonly in the 4th and 5th decades of life, on the weight bearing surface of femoral condyles or tibial plateau [14]. In the present study, osteochondral lesions caused by trauma, avascular necrosis (AVN), spontaneous necrosis (SPONK) and subchondral insufficiency fractures (SIF) were all classified in the Osteonecrosis group.

All the patients were evaluated routinely preoperatively, then at 12 and 24 months after surgery. At the time of the study (Last follow-up) a further assessment was performed.

At every examination, the following scores were collected: Visual Analog Scale (VAS) for pain [18], International Knee Documentation Committee (IKDC) Subjective Score, Tegner-Lysholm Knee Scoring Scale and Tegner Activity Level Scale [6]*.*

The minimal clinically important difference (MCID) was considered as 9.8 points for Subjective IKDC, 10.0 for Tegner Lysholm score and 1.5 for VAS score [32]. A substantial clinical benefit (SCB) was defined as difference between consecutive score evaluations greater than: 26.9 points for Subjective IKDC, 25.0 for Tegner Lysholm score and 2.3 for VAS score [36]. No reliable MCID and SCB values for Tegner Activity Level Scale were identified in literature.

Failure was defined, according to Filardo et al. [12], if one the following criteria were met: (1) clinical IKDC Subjective improvement inferior to 10 points at the maximum follow-up in comparison to the basal score or (2) the patient underwent revision surgery due to the persisting symptoms caused by the same lesion.

The study was approved by the scientific local board and all patients signed an informed consent to participate in the study.

### Scaffold features and indications

The 3D acellular scaffold (MaioRegen®) is composed of three layers of equine type I collagen and Magnesium-enriched hydroxyapatite (Mg-HA) nanocrystals in different concentrations. This structure supports the regeneration of the whole osteochondral unit providing a biomimetic environment for the cellular migration, adhesion and differentiation in bone tissue, tide-mark and articular cartilage. The porous scaffold is also totally biodegradable and biocompatible, fitting any lesion shape thanks to its flexibility [20, 21, 25, 27, 47].

This device is indicated as a surgical option in patients affected by a cartilaginous defects greater than 2.5 cm^2^ and graded III - IV according to ICRS classification [11], who did not show clinical improvements after 12 months of conservative treatment.

Contraindications to this treatment include: (1) uncorrected lower limb malalignment (> 5° varus or valgus knee), (2) kissing lesions, (3) unstable knee, (4) diffuse cartilage degeneration of the considered compartment (greater than grade II according to ICRS classification), (5) neoplastic or autoimmune or metabolic diseases, (6) previous knee localized infections, (7) BMI greater or equal to 30 or (8) skeletally immature patients.

### Surgical technique and post-operative care

**Fig. 1:**
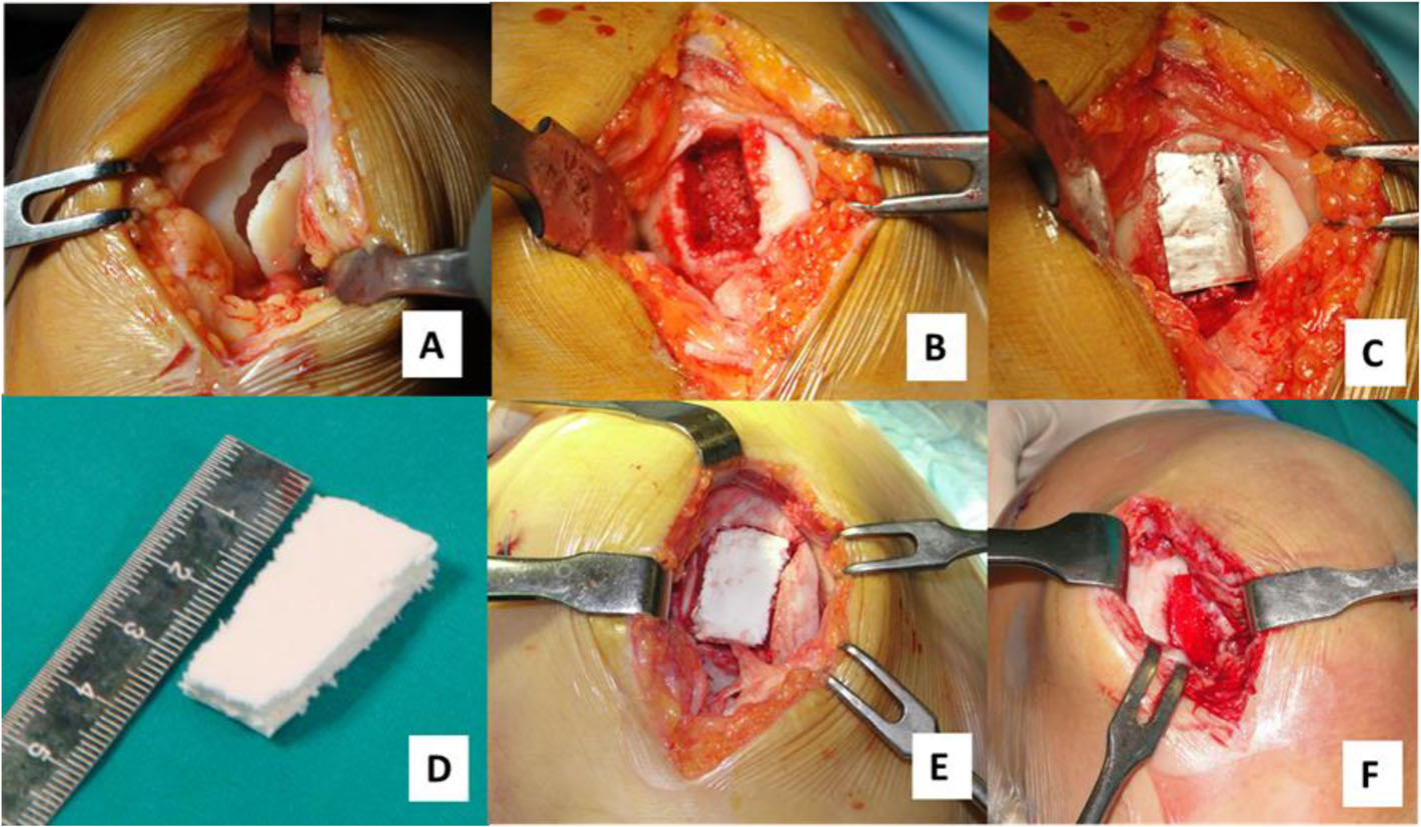
Surgical technique of biomimetic scaffold implantation in a medial femoral condyle osteochondral lesion. **a** Lesion exposure; **b** Implant site preparation (regular box-shape 9 mm deep); **c** Templating; **d** Scaffold sizing; **e** Press-fit implantation; **f** Bleeding from subchondral bone after tourniquet removal

All patients underwent osteochondral scaffold implantation by the same senior surgeon, experienced in knee cartilage repair procedures. The patient was positioned supine with the knee flexed at 90°. A tourniquet was applied and activated at the beginning of the arthrotomic procedure. Arthroscopic evaluation of the joint was performed to localize the defect and to examine the overall status of the joint. Both the defect and the surrounding cartilage were probed in order to estimate the margins of the lesion. A lateral or medial parapatellar approach, depending on the lesion localization, was carried out. The implant site was prepared removing the sclerotic subchondral bone and converting the lesion into a regular box-shaped defect with stable perimetral sides, always 9 mm deep in order to host the scaffold structure. A template was shaped to fit the prepared box and used to size the implant. Thereafter, the scaffold was implanted with a press-fit technique and its stability checked by bending the knee several times before and after the tourniquet removal (Fig. 1). Post-operatively, an intra-articular drain with no aspiration was left for 24 h in order to control excessive bleeding inside the joint.

An early 0–90° range of motion was allowed from the second day after surgery, even using continuous passive motion devices, and early isometric exercises were encouraged. Toe-touch weight-bearing with two crutches was allowed from the second postoperative day using a brace in full extension. Starting from the 3rd to the 6th postoperative week, weight-bearing was gradually increased to 15–20 Kg and knee flexion raised to 120°, according to the knee local conditions and patient compliance. At 6 weeks, the patient was encouraged to progressively reach full weight-bearing and both crutches and brace were dismissed at 8 weeks after surgery. Water assisted exercises and low resistance bicycle were introduced, while squatting was discouraged until 3 months post-operatively. Sports activities were allowed not earlier than 10 months after the operation and only in asymptomatic patients who completely restored muscle strength (90% compared to the contralateral limb in isokinetic tests).

### Statistical analysis

All the analyses were performed using SPSS Statistics for Windows, Version 20.0 (IBM Corporation, Armonk, NY, USA). The Kolmogorov-Smirnov test was used to assess normal data distribution. A two-way repeated-measures ANOVA test for within-subject factors (time) as well as between-subjects factors (sex, age lower or greater than 35 years, aetiology) with Bonferroni correction was performed in order to assess differences between each follow-up measures. The following variables were analysed for within-subject factors as well as between-subjects factors: Subjective IKDC, Tegner-Lysholm and VAS scores, and Tegner Activity Level Scale. Statistical significance was achieved if *p* < 0.05.

## Results

Forty-five patients were screened for eligibility and 29 of them (64%) were enrolled in the current study according to the inclusion-exclusion criteria (23 males and 6 females). Four patients (9%) were excluded because of previous surgical treatment on the same knee, while 12 patients (27%) did not reach the minimum follow-up of 5 years after the scaffold implantation. Mean age at the surgery time was 31.5 ± 15.4 years and mean follow-up 7.8 ± 2.0 years (range 5.1–11.3 years).

The underlying etiology was Osteochondritis Dissecans (OCD) in 17 patients (58.6%) and osteonecrosis (ON) in 12 cases (41.4%). Demographic and lesion related data are reported in Table 1.

**Table 1:** Demographic and lesion related data

	Value
Gender, Male/Female (n)	23/6
Injured side, right/left (n)	13/16
Age at the surgery (years) - Mean value ± SD (Range)	31.5 ± 15.4 (15.6–65.2)
Age, ˂35/≥ 35 years (n)	18/11
Aetiology, OCD/ON (n)	17/12
Localization, MFC/LFC (n)	25/4
BMI (kg/m^2^)	24.3 ± 2.1
Lesion area (cm^2^)	3.8 ± 0.8
Follow-up (years) - Mean value ± SD (Range)	7.8 ± 2.0 (5.1–11.3)

*Abbreviations:OCD* osteochondritis dissecans, *ON* osteonecrosis, *MFC* medial femoral condyle, *LFC* lateral femoral condyle, *BMI* body mass index

Compared to the preoperative values, at 12 months follow-up the IKDC score changed from 51.1 ± 21.7 to 80.1 ± 17.9 (*p* < 0.01), Tegner Lysholm Score from 59.9 ± 17.3 to 92.5 ± 9.0 (*p* < 0.01), VAS score from 6.1 ± 2.1 to 1.7 ± 2.3 (*p* < 0.01) Most of patients were able to return to sport as proved by the Tegner Activity Level Scale improvement from 1.6 ± 0.5 pre operatively to 4.9 ± 1.7 at 1 year (*p* < 0.01). Moreover, all the clinical scores showed an improvement that exceeded their relative MCID and SCB threshold values.

**Fig. 2:**
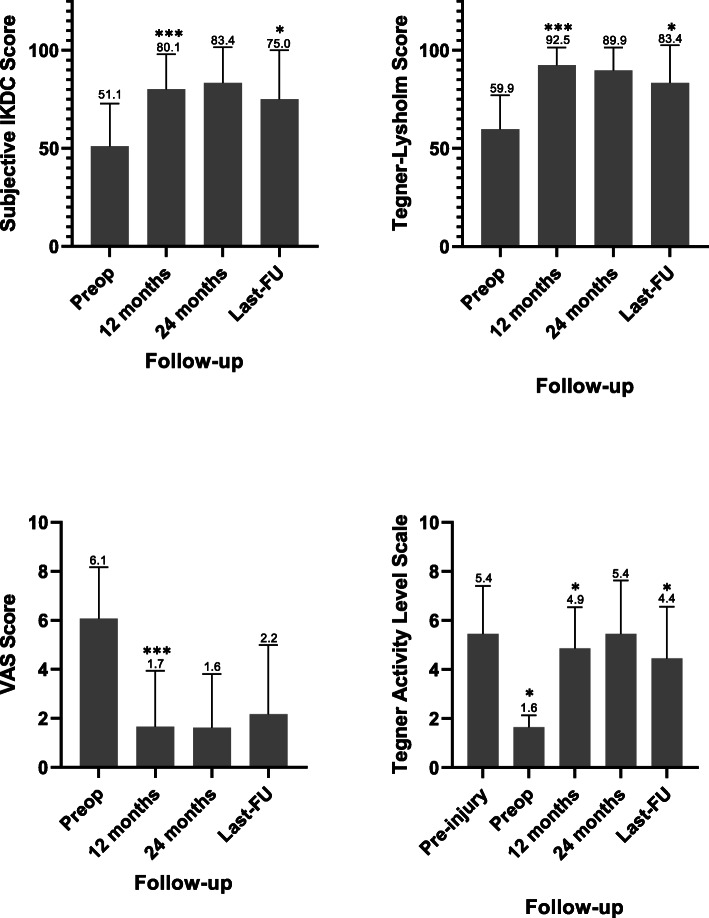
Outcome evaluation. Mean value of the clinical scores collected preoperatively, at 12–24 months after surgery and last follow-up. (* = statistically significant difference, ** = statistically significant difference and minimal clinically important difference, *** = statistically significant difference and minimal clinically important difference and subjective clinical benefit)

Considering the trend between 24 months and last-FU, a statistically significant decrease in Subjective IKDC, Tegner Lysholm Score and Tegner Activity Scale was recorded, respectively from 83.4 ± 18.1 to 75.0 ± 25.0 (*p* < 0.05), from 89.9 ± 11.6 to 83.4 ± 19.2 (*p* < 0.05), and from 5.4 ± 2.2 to 4.4c2.1 (*p* < 0.05). These differences, though, resulted lower than the corresponding MCID values. VAS score, instead, did not show any statistically significant variation in this time interval, achieving a mean value of 2.2 ± 2.8 at the last check (Fig. 2).

**Fig. 3:**
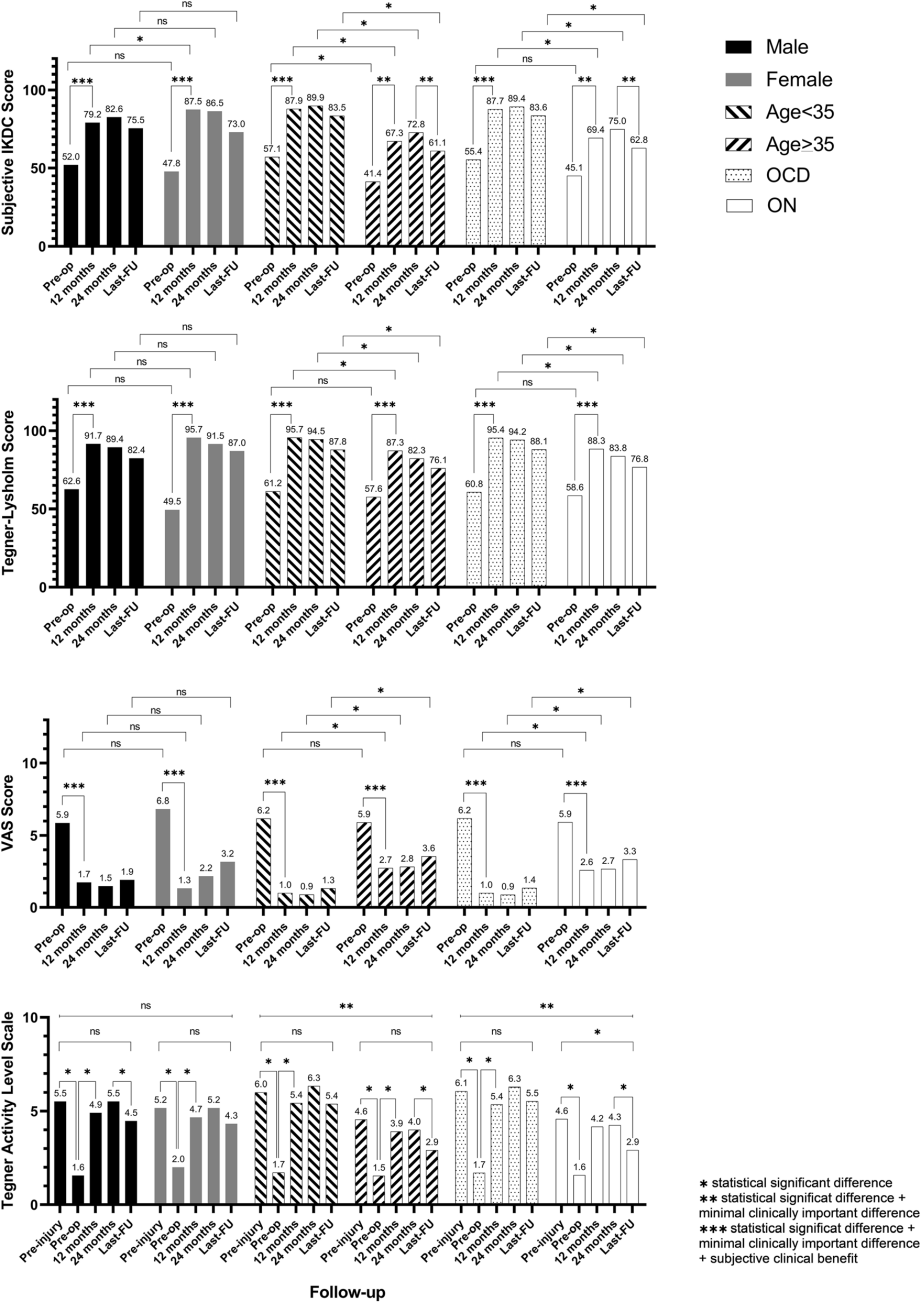
Subgroups analysis. Analysis of data depending on gender (Male/Female), age (< 35/> 35 years) and lesion etiology (Osteochondritis Dissecans/Osteonecrosis) for Subjective IKDC Score, Tegner-Lysholm score, VAS and Tegner Activity Level Scale (NS = not statistically significant)

Patients under 35 years of age and the ones affected by Osteochondritis Dissecans achieved statistically better outcomes at every follow-up, whereas sex did not influence the results. Details about the influence of sex, age and lesion aetiology on the results and the trend of very analysed score are reported in Fig. 3. Any improvement which exceeded the MCID and SCB was also marked to highlight the clinically relevant achievement (Fig. 3).

**Fig. 4:**
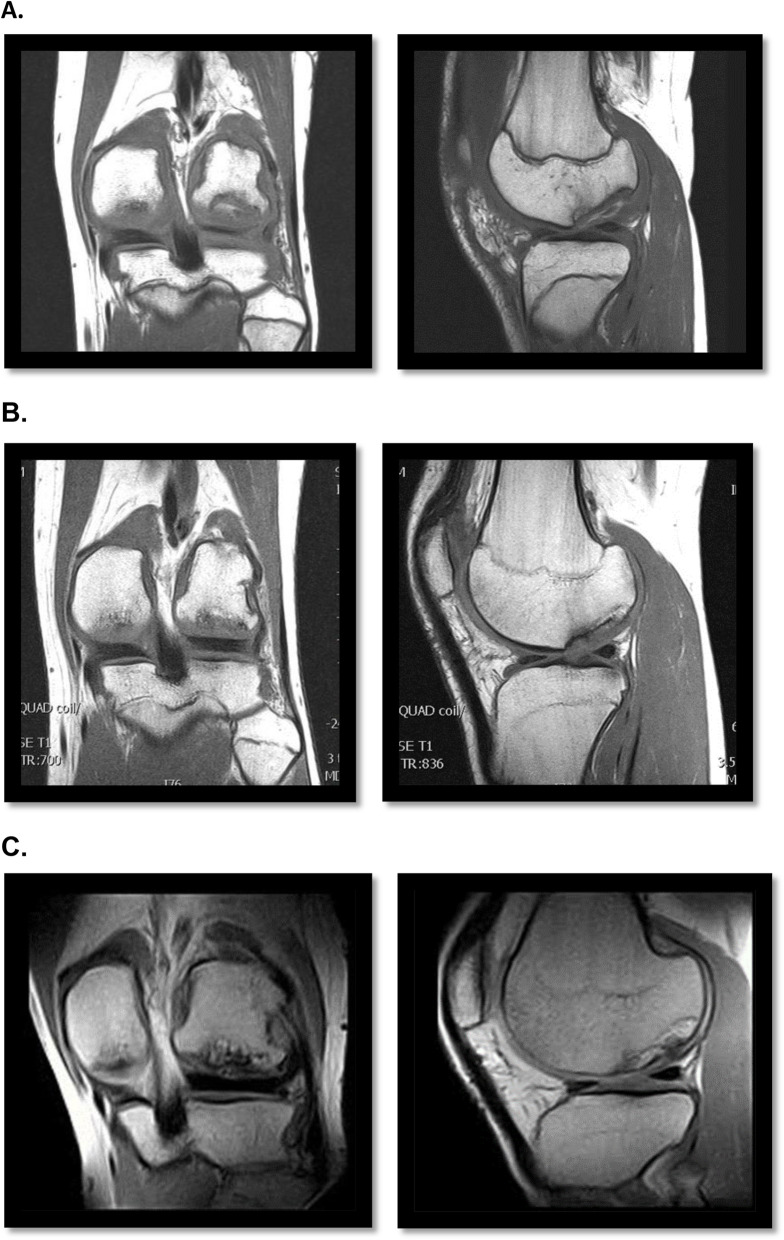
Sample case. Magnetic resonance imaging (coronal and sagittal view) of an osteochondritis dissecans on the lateral femoral condyle of the left knee in a 15 years old male treated with Maioregen® osteochondral scaffold. **a** Pre-operative; **b** 12 months follow-up; **c** 5 years follow-up

In Fig. 4 an example of the evolution on MRI of osteochondritis dissecans after the scaffold implantation is reported, showing a good integration of the device and excellent restoration of the articular surface (Fig. 4).

No intraoperative or postoperative major complications (such as infection, wound dehiscence, thromboembolism, nerve or vessel injuries) were recorded.

Five patients were identified as failures, 4 men and 1 woman (mean age 46.6 ± 14.7 years). In 3 cases the failure occurred 1 year post-operatively, while 2 patients reported a severe worsening of symptoms respectively 4 and 8 years after surgery. All the failures involved lesions located on the medial femoral condyle, whereas the underlying etiology was ON in 3 cases and OCD in 2 cases.

## Discussion

The main finding of the current study was that the treatment with a cell-free osteochondral scaffold resulted in substantial clinical benefits in isolated osteochondral defects of the knee and these results were maintained at a follow-up greater than 5 years, thus our hypothesis was confirmed. A clinical improvement was observed regardless gender, age or lesion etiology, although higher outcomes were noticed especially in patients younger than 35 years or affected by osteochondritis dissecans. To the best of our knowledge, the current study represents one of the largest case series at mid-term follow-up available in literature with a mean follow-up of 7.8 years, ranged 5.1 to 11.3 years.

Several strategies for treating large cartilaginous lesions have been introduced in clinical practice with controversial indications and results; therefore, an agreement about the most effective technique is yet to be found. Microfractures, despite excellent short-term results [28, 29, 34, 35], demonstrated results deterioration at 5 years when compared to advanced cartilage repair techniques [16]. This difference gets even more significant at longer follow-up (10 years) [33, 39], leading to a high reoperation rate. Matrix-assisted chondrocytes transplants were developed in order to overcome microfractures limitations and showed stable clinical result up to 2 years [1, 41]. Good to excellent results have been reported using ACI techniques in OCD at both mid and long-term evaluation [5, 13], despite being burdened with high costs for cells culture and the need of two surgical procedures. The recent identification of the role of the subchondral bone in cartilage regeneration has led to a surgical approach focused on the treatment of both cartilage and subchondral bone [30]. Osteochondral autografts or mosaicplasty are alternative solutions in case of deep defects but they cause a donor site morbidity and in some cases they cannot fill the defect perfectly [17]. Allografts are useful in large osteochondral injuries or revision surgery [15], but they are not easily available in all centers and carry the risk of infections.

In light of this evidence, osteochondral cell-free scaffolds represent a single-step and morbidity free alternative in the treatment of severe osteochondral lesions, with the aim of fully restoring the osteochondral unit. In the current study, a three-layered acellular scaffold was used.

Previous studies at short term follow-up have been published reporting clinically significant results at 12, 24 and 36 months [8, 26, 31, 37], supporting the positive outcomes of this technique in the treatment of osteochondral defects. Collagen-hydroxyapatite scaffolds provided a significant clinical improvement in 20 patients affected by unfixable osteochondritis dissecans at 6 years follow-up, showing durable benefits and return to sport [42]; the same implant was used in 22 cases of early osteoarthritis with a satisfactory clinical outcome and a low failure rate at 60 months after surgery [41], moreover this technique has shown to be an option also in late-stage osteonecrosis of the knee, as an alternative to early arthroplasty in relatively young and active patients [2]. In a Multicenter Randomized Trial by Kon and coauthors [23], cell-free scaffolds were compared to bone marrow stimulation techniques (BMS) for treating osteochondral lesions at a minimum follow-up of 2 years: no overall statistically significant differences were detected between the two treatments in term of clinical outcomes, but patients presenting deeper osteochondral lesions demonstrated statistically significant higher IKDC Subjective values (+ 12.4 points, *p* = 0.036) in the scaffold group compared to BMS. In the present study, instead, the lesion depth was not calculated because all the analyzed defects were full thickness osteochondral lesions.

Perdisa et al. [37] reported about 27 patients affected by OCD who underwent osteochondral scaffold implantation evaluated prospectively every 12 months for 5 years. The author reported a significant IKDC score improvement from baseline at 12 and 24 months, which was maintained at 60 months follow-up (from 48.4 ± 17.8 to 90.1 ± 12.0). A comparable improvement in Tegner Activity Level scale, from 2.4 ± 1.7 to 5.0 ± 1.7, at 60 months follow-up was reported too. On the other hand, in our study, overall inferior results were recorded and a statistically significant decrease in both IKDC and Tegner Lysholm score were found, despite inferior to the MCID. The difference in patients reported outcome could rely on the different population object of the analysis. Indeed, in our analysis both OCD and ON lesions were considered, respectively 17 and 12 cases, and patients up to 65 years old were included. Considering that the IKDC score highly relies on sport-like activities, a decrease in the performance of demanding physical exercise related to aging could negatively affect outcome scores resulting in lowers values in comparison to a younger population. This hypothesis can be supported by the data obtained from the analysis of OCD patients only, resulting in values which almost overlap the ones proposed by Perdisa et al. [37].

In another study from the same group [22], Kon and co-authors reported the results of 27 patients (5 traumatic, 16 degenerative and 6 OCDs) prospectively evaluated at 24 and 60 months after scaffold implantation. A statistically significant increase in IKDC subjective score that lasted up to last follow-up was observed, from 40.0 ± 15.0 at baseline to 76.5 ± 14.5 (2 years FU) and 77.1 ± 18.0 (5 years). A lower Tegner Activity Scale value was described at 5 years in comparison to the pre-injury level, albeit non-significant. In this series, aetiology, sex and age were not correlated to the final outcomes. In the current study both age over-35 and osteonecrosis aetiology negatively affected both absolute values and data trend in patients reported outcomes. A statistically significant decrease in IKDC score was observed at last-FU in both patients aged over 35 and ON group, despite inferior to the cut off value proposed by Ogura et al. to define a subjective clinical benefit [35]. Nevertheless, no significant increase in VAS score was assessed considering independently the two groups. Similarly, in a study about 23 patients affected by Early Osteoarthritis treated with a cell-free scaffold, Di Martino et al. [10] reported a significant improvement in knee function at 1 year, but found a difference between patients under vs over 40 years of age, with younger patients achieving better clinical scores (76.0 ± 18.6 vs 45.1 ± 38.8 respectively, *p* = 0.037). Therefore, despite patients in the Osteonecrosis group in our series achieved a worse outcome, still not clinically significant, the treatment with the biomimetic scaffold can represent an alternative solution to joint replacement in young and active patients with an isolated osteochondral lesion, as suggested in previous studies [2, 43].

Conversely, the clinical scores investigated in the present study have shown a better improvement compared to the pre-operative values, especially in young patients and OCD lesions. Our results are in line with the ones reported by Kon et al. [24] who noticed that patients affected by OCDs had a higher IKDC Subjective Score than those with degenerative lesions at 2 years follow-up (*p* = 0.035) and confirm this population can probably benefit the most from scaffold implantation.

Concerning the slight decline in Subjective IKDC, Tegner Lysholm Score and Tegner Activity Scale recorded at last follow-up compared to the results at 2 years after surgery, the authors ascribe these findings to the natural decrease in participation in high demanding physical activities, included sport, with the raising age of the enrolled population, which was mostly due to changes in lifestyle and not to knee-related symptoms. This theory is confirmed by VAS values that show a stable effect on pain several years after the procedure. A physiological degenerative process could also affect the operated joint as often described after any cartilage repair technique, thus leading to a slow deterioration in functional outcome at long term.

Among the limitations of this study, we acknowledge its retrospective nature, the lack of a control or comparative group and the limited number of patients enrolled, which is caused by the strict including criteria. Furthermore, results regarding postoperative MRI and X-Rays were not reported due to the lack of available postoperative imaging at the last-FU.

A longer follow-up and a larger cohort of patients would be necessary to confirm the durability of clinical results, the survival rate of the implant and potentially the eventual onset of degenerative changes.

## Conclusion

A cell-free osteochondral scaffold provided a significant improvement in clinical outcome compared to the preoperative values and the results were maintained at over 5 years of follow-up. Patients were able to return to the same activity level as before the injury occurred. The best clinical outcome was achieved in young patients and OCD. These findings confirm that the use of a biomimetic scaffold is a reliable option in the treatment of knee osteochondral lesions and OCD at medium-term follow-up.

## Data Availability

The datasets used and/or analyzed during the current study are available from the corresponding author on reasonable request.
